# A Comprehensive Review and Perspective on Natural Sources as Dipeptidyl Peptidase-4 Inhibitors for Management of Diabetes

**DOI:** 10.3390/ph14060591

**Published:** 2021-06-20

**Authors:** Sibhghatulla Shaikh, Eun-Ju Lee, Khurshid Ahmad, Syed-Sayeed Ahmad, Jeong-Ho Lim, Inho Choi

**Affiliations:** 1 Department of Medical Biotechnology, Yeungnam University, Gyeongsan 38541, Korea; sibhghat.88@gmail.com (S.S.); gorapadoc0315@hanmail.net (E.-J.L.); ahmadkhursheed2008@gmail.com (K.A.); sayeedahmad4@gmail.com (S.-S.A.); lim2249@naver.com (J.-H.L.); 2 Research Institute of Cell Culture, Yeungnam University, Gyeongsan 38541, Korea

**Keywords:** diabetes, dipeptidyl peptidase-4, medicinal plants, natural compounds

## Abstract

Type 2 diabetes mellitus (T2DM) is an increasing global public health problem, and its prevalence is expected to rise in coming decades. Dipeptidyl peptidase-4 (DPP-4) is a therapeutic target for the management of T2DM, and its inhibitors prevent the degradation of glucose-dependent insulinotropic peptide and glucagon-like peptide 1, and thus, maintain their endogenous levels and lower blood glucose levels. Various medicinal plant extracts and isolated bioactive compounds exhibit DPP-4 inhibitory activity. In this review, we discussed different natural sources that have been shown to have anti-diabetic efficacy with a particular emphasis on DPP-4 inhibition. Furthermore, the effect of DPP-4 inhibition on pancreatic beta cell function, skeletal muscle function, and the glucose-lowering mechanisms were also discussed. We believe that scientists looking for novel compounds with therapeutic promise against T2DM will be able to develop antidiabetic drugs using these natural sources.

## 1. Introduction

Type 2 diabetes mellitus (T2DM) is a metabolic condition marked by a prolonged hyperglycemic state caused by a combination of underlying defects, which include insulin tolerance in muscle and liver, and reduced insulin production by pancreatic beta cells [1,2]. T2DM is the most prevalent form of diabetes and accounts for about 91% of all cases, and the disease has been predicted to affect about 366 million people by 2030 [3]. T2DM is marked by enhanced blood glucose (BG) levels and microvascular and macrovascular complications that substantially enhance disease-associated morbidity and mortality, and epidemiological data show that people with diabetes are at slightly higher risk of developing various types of cancer, and musculoskeletal, cardiovascular, and psychiatric disorders [4,5,6,7]. Dipeptidyl peptidase-4 (DPP-4) has emerged as a target in T2DM, and as a result, its inhibitors are attracting increased research interest. DPP-4 accelerates the degradations of the incretin hormones glucagon-like peptide 1 (GLP-1) and glucose-dependent insulinotropic peptide (GIP) by removing a dipeptide from their N termini, resulting in altered glucose homeostasis. Interestingly, DPP4 knockout mice are resistant to diet-induced obesity and have better postprandial glucose balances than their counterparts [8,9].

Insulin controls the metabolism of carbohydrates, fats, and proteins [10], and thus, any defects in insulin synthesis or its activities cause severe metabolic complications. Insulin is produced by pancreatic beta cells, and as T2DM progresses, beta cell functions decline due to rising hyperglycemia and insulin resistance. This cellular decline can start early during the course of T2DM and worsens due to compensatory overload, which accelerates disease progression. Beta cell dysfunction results from deficient glucose sensing, and thus, insulin release, which increases glucose concentrations [11,12].

Many plants have been used to treat diabetes, and interest in medicinal plants as a source of medicines has increased [13,14]. Herbal medicines are considered to importantly complement oral hypoglycemic agents for the management of T2DM, and to have played important roles in the management of diabetes in several countries by preventing diabetic complications and fixing metabolic irregularities [15,16]. We undertook the present review to provide an update on the latest advances made to develop DPP-4 inhibitors derived from natural resources.

## 2. Dipeptidyl Peptidase-4

DPP-4 (also termed cluster of differentiation 26, CD26) is a serine exopeptidase, a 220 kDa homodimeric type II transmembrane glycoprotein, found on the surfaces of different cell types. DPP-4 cleaves X-proline dipeptides from polypeptides including chemokines, neuropeptides, and peptide hormones at their N-termini, and is expressed in a variety of tissues, including endothelial cells in various vascular beds, which makes it particularly accessible to peptide substrates in gut, stomach, kidney, and liver [17]. The DPP4 gene encodes a 766-amino-acid protein and is located on chromosome 2q23 in man [18]. After being synthesized, DPP4 is immediately integrated into plasma membranes. It is a type II surface protein, which means most of the structure, including its C-terminal domain, is located in the extracellular domain. However, DPP4 can be released from the membrane in response to certain stimuli, such as insulin resistance, tumor necrosis factor-alpha, and chronic low-grade inflammation, resulting in its soluble form [19].

## 3. Incretin Hormones

**Figure 1:**
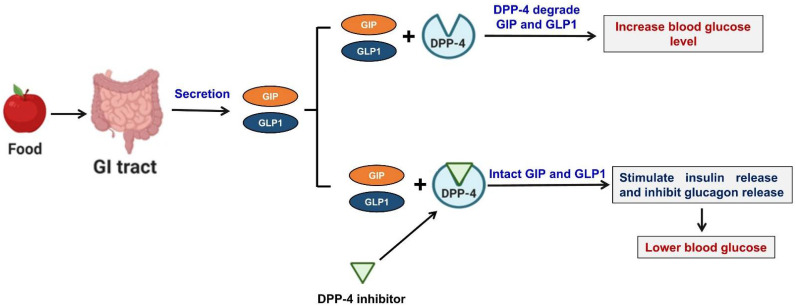
Proposed mechanism of DDP-4 inhibition. In response to nutrient intake and/or an enhanced BG level, incretins (GLP-1 and GIP) are released from the gastrointestinal tract. These two incretins enhance insulin synthesis and secretion and inhibit the release of glucagon, and thus, reduce BG levels in healthy individuals. However, in T2DM, DPP-4 rapidly degrades both incretins and renders them inactive. DPP-4 inhibitors act by preventing DPP-4-induced incretin degradation, increasing intact GLP-1 and GIP levels, and improving glucose homeostasis.

Incretins are a class of metabolic hormones that stimulate a drop in BG levels. Incretin deficiency/resistance plays a vital role in the progression of T2DM. GLP-1 and GIP are the two main human incretins that control the maintenance of glucose homeostasis. GLP-1 is released by intestinal endocrine L-cells, which are often found in ileum and colon, whereas GIP is secreted by intestinal K-cells in the more proximal regions (duodenum and jejunum) of the small intestine [20]. In response to nutrient consumption and/or enhanced BG levels, GLP-1 and GIP are released from the gastrointestinal tract. These two incretins enhance the action of insulin, inhibit the release of glucagon, and reduce liver glucose production, which in healthy individuals lowers BG levels. GLP-1 and GIP are the best-characterized DPP-4 substrates in terms of metabolic effects. In T2DM, endogenous GLP-1 is quickly degraded by DPP-4, and thus, its insulinotropic function is lost. On the other hand, preventing this degradation results in higher GLP-1 levels and enhanced pancreatic islet response, and improved glucose homeostasis. DPP-4 also effectively cleaves GIP, and inhibiting DPP-4 enhances GIP levels and its effects [20]. Therefore, DPP-4 is viewed as an important therapeutic target for the management of T2DM (Figure 1).

## 4. Commercialized DPP-4 Inhibitors for the Treatment of Diabetes

GLP-1 and GIP both control insulin release in a glucose-dependent manner. However, endogenous GLP-1 and GIP have plasma half-lives of ~7 and 1 to 2 min, respectively, due to their rapid enzymatic deactivations by DPP-4. The biological activities of these two peptides are determined by the Xaa-pro and Xaa-ala sequences, which also act to prevent non-specific proteolysis [21,22]. Several DPP-4 inhibitors, such as gliptin, are currently approved for the treatment of T2DM. The first DPP-4 inhibitor approved by the FDA was sitagliptin [23], which was followed by vildagliptin [24], saxagliptin [25], alogliptin [26], and linagliptin [27]. More recently, the following inhibitors were approved; anagliptin [28], gemigliptin [29], and teneligliptin [30] in 2012; evogliptin [31], omarigliptin [32], and trelagliptin [33] in 2015; and gosogliptin [34] in 2016. While their binding characteristics and pharmacokinetic properties vary, all DPP-4 inhibitors are orally active, selective for DPP-4, and have a high affinity for the enzyme [35]. Table 1 lists commercialized DPP-4 inhibitors along with their brand name and approval year.

## 5. DPP-4 Inhibition and Pancreatic Beta Cell Function

DPP-4 antagonists provide long-term, reliable, and effective treatments for T2DM that provide strong glycemic control. GLP-1 and GIP act on G-protein coupled receptors, which are expressed on pancreatic beta and alpha cells and in peripheral tissues, to lower glucose levels [20]. GLP-1 enhances insulin secretion, insulin gene transcription, and insulin biosynthesis by acting on pancreatic beta cells [36].

T2DM causes a gradual reduction in beta cell activity, and thus, to reverse insulin secretory defects, beta cell activity must be restored. Given that GLP-1 has been reported to induce the proliferation and inhibit the apoptosis of beta cells in rodents and to induce beta cell differentiation from human precursor cells [37,38], it seems safe to assume that DPP-4 inhibition enhances GLP-1 levels, and thus, improves beta cell mass and survival. Animal studies have shown that DPP-4 inhibitor promotes islet neogenesis, beta cell regeneration, and/or improved insulin biosynthesis, and thereby preserves or increases beta cell numbers [37,39]. Accordingly, histological analysis of pancreases after DPP-4 inhibitor treatment revealed elevated numbers of islets and beta cells [37].

DPP-4 inhibition has been shown to reduce T2DM-induced beta cell dysfunction and apoptosis in in vitro and in pre-clinical studies. DPP-4 is present in mouse and human islets, and inhibiting islet DPP-4 activity has been shown to have a direct stimulatory effect on GLP-1-dependent insulin secretion [40,41]. A similar effect was demonstrated in db/db diabetic mice after 2 weeks of des-F-sitagliptin treatment, which resulted in enhanced insulin exocytosis by beta cells [42]. In addition, DPP-4 inhibition has been related to beta cell mass and functional increases in several T2DM models [43,44], and the transcriptional activations of anti-apoptotic and pro-survival genes have also been linked to these positive effects in beta cells [45]. Furthermore, the DPP-4 inhibitor linagliptin has been shown to protect isolated human islets from gluco- and lipotoxicity [46], and interestingly, vildagliptin has been shown to have antioxidant properties, as evidenced by dose-dependent reductions in nitric oxide concentrations in serum and pancreatic homogenates of diabetic rats [47].

## 6. DPP-4 Inhibitors Improve Blood Glucose Response

DPP-4 inhibitors have been shown to decrease BG levels in T2DM patients by continuous glucose monitoring (CGM) [48,49,50]. In addition, various randomized controlled trials have shown by CGM that DPP-4 inhibitors suppress BG levels more efficiently than other agents such as sulfonylureas [51,52] or sodium glucose cotransporter 2 inhibitors [53,54,55] when used in combination with insulin administration [56,57]. DPP-4 inhibitors that enhance insulin secretion and decrease prandial glucagon levels have been shown to improve BG levels [49], and reductions in prandial glucagon levels are considered to underlie improvements in BG levels by DPP-4 inhibitors [58]. Vildagliptin was reported to lower postprandial glucagon levels and improve hyperglycemia in T2DM patients [58], and DPP-4 inhibitors were observed to increase the abilities of alpha and beta cells to detect and respond to hypoglycemia [59]. Furthermore, DPP-4 inhibitors can improve both hyperglycemia and hypoglycemia [60,61]. Moreover, when DPP-4 inhibitors block persistent glucagon oversecretion [58], glucagon responds normally to a drop in BG level and ameliorates hypoglycemia [59]. Overall, these studies show that DPP-4 inhibitors can improve hypoglycemia/hyperglycemia and BG levels in T2DM patients.

## 7. DPP-4 Inhibition and Skeletal Muscle Function

Skeletal muscles (SM) comprise the largest organ in the body, and thus, process the largest amounts of administered drugs [62]. Furthermore, muscle has recently been reported to release DPP-4 [63]. SM cell cultures were observed to release DPP-4 during differentiation [64], and DPP-4 activity in the bathing medium from intact SM was found to be enhanced by whey protein in situ [65]. DPP-4 inhibitors were also found to reduce SM loss in T2DM patients [66]. Recently, sitagliptin was reported to increased muscle mass and muscle/fat ratio in T2DM [67], and natural DPP-4 inhibitors such as chrysin and galangin were reported to promote SM cell proliferation [68,69]. Furthermore, in a diabetic animal model, myricetin administration reduced DDP-4 expression in muscle [70]. Overall, these studies revealed that DPP-4 inhibitors have a positive effect on SM, increasing SM cell proliferation and the muscle/fat ratio in diabetic patients.

## 8. DDP-4 Inhibitors from Natural Sources

Nature is a plentiful source of medicinal herbs, and several natural foods that contain bioactive components with health benefits are commonly used as herbal remedies for many life-threatening diseases [71,72,73]. Herbal remedies offered a valuable resource for pharmacological agents for diabetes even before insulin and other pharmacological drugs were discovered, and have become an increasingly important aspect of searches for curative and adjunctive treatments [74]. Below, we detail plants with extracts that inhibit DPP-4 and their corresponding IC_50_ values; a summary is also provided as a list in Table 2.

### 8.1. Urena lobata

*Urena lobata* (Caesar weed or Congo jute) is a traditional herb found in many countries and has promising biological activities. *U. lobata* root extract had antihyperglycemic effects on streptozotocin-induced diabetic rats [75], and in vitro, an ethanolic extract of *U. lobata* showed 4-fold greater DPP-4 inhibitory activity (IC_50_ = 1.65 mg/mL) than water extract (IC_50_: 6.49 mg/mL) [76].

### 8.2. Anogeissus latifolia and Aegle marmelos

*Anogeissus latifolia* and *Aegle marmelos* are members of the Combretaceae and Rutaceae families, respectively, and are used traditionally to treat diabetes, hemorrhages, diarrhea, asthma, dysentery, skin diseases, leprosy, and hepatopathy [77,78]. *A. latifolia* and *A. marmelos* extracts inhibited DPP-4 with IC_50_ values of 754 and 790 µg/mL, respectively, and improved glucose homeostasis and insulin release in high-fat diet (HFD)-diabetic rats [79].

### 8.3. Castanospermum austral

*Castanospermum austral* (also called black bean) is an herb that grows in Australian coastal regions and rainforests. *C. australe* seed extract inhibited DPP-4 with an IC_50_ of 13.96 µg/mL, while the control compound diprotin A had an IC_50_ of 1.543 µg/mL. In addition, in a T2DM animal model, *C. australe* seed extract lowered BG levels, prevented hyperinsulinemia, and increased glucose tolerance [80].

### 8.4. Fagonia cretica and Hedera nepalensis

*Fagonia cretica* (FC) belongs to the Zygophyllaceae (Caltrop) family, and *Hedera nepalensis* is a member of the family Araliaceae and is found in Nepal and Bhutan, Afghanistan, Pakistan, India, China, Myanmar, Thailand, and Vietnam. The crude extracts of FC and *H. nepalensis* strongly inhibited DPP-4 with IC_50_ values of 38.1 and 17.2 μg/mL, respectively. Four compounds (quinovic acid, quinovic acid-3β-*O*-β-d-glycopyranoside, quinovic acid-3β-*O*-β-d-glucopyranosyl-(28→1)-β-d-glucopyranosyl ester, and stigmasterol) isolated from FC had IC_50_ values of 30.7, 57.9, 23.5, and >100 µg/mL, respectively [81].

### 8.5. Eugenia jambolana and Pterocarpus marsupium

*Eugenia jambolana* is an evergreen, tropical, fruit-producing tree found in South Asia and South America, while *Pterocarpus marsupium* is native to India, Nepal, and Sri Lanka. Both *P. marsupium* and *E. jambolana* had potent inhibitory effects on DPP-4 with IC_50_ values of 273.73 and 278.94 µg/mL, respectively [82].

### 8.6. Chenopodium quinoa Willd

Quinoa (*Chenopodium quinoa Willd*) is a flowering plant of the amaranth genus Amaranthaceae. Quinoa is a gluten-free grain that has a greater protein content than other grains including wheat, rice, maize, oat, and barley. Analysis of quinoa protein hydrolysate revealed potent DPP-4 inhibitory activity (IC_50_ 0.88 mg/mL) [83].

### 8.7. Allium sativum

*Allium sativum* (garlic), a member of the Alliaceae family, is widely used as a spice and as a treatment for a variety of diseases and physiological conditions [84]. Its bulb extract inhibits DPP-4 activity (IC_50_ 70.9 µg/mL) and enhances SM cell proliferation [85].

### 8.8. Pilea microphylla

*Pilea microphylla* (the gunpowder plant) is an annual herb found in Florida, Mexico, and tropical Central and Southern America. In vitro, *P. microphylla* inhibited DPP-4 with an IC_50_ of 520.4 µg/mL. In addition, in an HFD/streptozotocin-induced diabetic rat, *P. microphylla* reduced plasma glucose and prevented beta cell destruction [86].

### 8.9. Mangifera indica

*Mangifera indica* (MI) is an ayurvedic herb that belongs to the Anacardiaceae family. MI leaf extract has been shown to have hypoglycemic properties [87]. The extract of its leaves was tested in vitro for DPP-4 inhibitory activity, and the results reveal an IC_50_ of 182.7 µg/mL [88]. The main phytochemical in MI is mangiferin. In HFD/streptozotocin-induced diabetic rat, lower serum DPP-4 levels were associated with improved insulin resistance and improved beta cell function [89].

### 8.10. Lilium longiflorum

*Lilium longiflorum* (Liliaceae) bulbs are used as food ingredients and herbal medicines in East Asia. Treatment with the ethyl acetate fraction of *L. longiflorum* was shown to inhibit DPP-4. Five compounds were purified from the ethyl acetate fraction of *L. longiflorum*, and compounds **2** and **5** were found to exhibit DPP-4 inhibitory activity with IC_50_ values of 46.19 and 63.26 µM, respectively [90].

### 8.11. Coreopsis lanceolata

*Coreopsis lanceolata* is a perennial herb of the Compositae family. A methanol extract of the flowers of *C. lanceolata* was found to inhibit DPP-4 activity by 87.2%. Among the various compounds isolated, compounds **2**–**4**, **6**, and **7** inhibited DPP-4 in a concentration-dependent manner, with IC_50_ values ranging from 9.6 to 64.9 µM [91], which suggests that flowers of *C. lanceolata* and their active components have potential for the treatment of T2DM.

### 8.12. Psidium guajava *L*.

*Psidium guajava* L. (Guava) is a member of the Myrtle family (Myrtaceae). Guava leaves have a long history of use in traditional and conventional medicine that spread from South America to tropical Asia and Africa. Ethanolic guava leaf extract (IC_50_ 380 μg/mL) and flavonol glycosides isolated from the extract inhibited DPP-4 in a dose-dependent manner [92].

### 8.13. Melicope glabra

*Melicope glabra* is a tree of the Rutaceae family herb and an important source of flavonoids and coumarins. The plant is native to Sumatra, Peninsular Malaysia, Singapore, Java, and Borneo. The chloroform extract of the leaves of *M. glabra* effectively inhibited DPP-4 with an IC_50_ of 169.40 μg/mL. Computational analysis showed that compounds (**8**) and (**7**) in this extract are potent DPP-4 inhibitors based on their binding affinities and extensive interactions with important DPP-4 residues [93]. The phytochemical profiles of these compounds indicated their potential as DPP-4 inhibitors.

### 8.14. Hibiscus rosa-sinensis

*Hibiscus rosa-sinensis* (HRS) is a tropical flowering plant that is common in Asia and is used in herbal medicine to treat a variety of ailments, such as cough, diarrhea, and diabetes. An ethanol extract of HRS significantly inhibited DPP-4 activity, increased insulin release, and thus, improved glucose tolerance in type 2 diabetic rats [94].

### 8.15. Annona squamosa

*Annona squamosa,* commonly called ‘Ata’, is a small tree that belongs to the Annonaceae family and is native to Bangladesh. This herb is well known for its various medicinal properties, which include antioxidant, anti-diabetic, and hepatoprotective effects [95]. Hot water extract of *A. squamosa* was recently reported to promote cellular glucose absorption and the secretion/action of insulin, and to suppress DPP-4 activity [96].

### 8.16. Emblica officinalis

*Emblica officinalis*, commonly known as Indian gooseberry (amla), is a member of the Phyllanthaceae family and used as a folk medicine to treat various diseases, including diabetes. Amla fruit extract inhibited DPP-4 (IC_50_ 3770 μg/mL) and alpha-glucosidases and exhibited antioxidant properties [97].

### 8.17. Berberis aristata

*Berberis aristata* belongs to the Berberidaceae family and is a shrub native to the Himalayas in India and Nepal. The roots of this plant have antibacterial, anti-inflammatory, analgesic, antioxidant, and hepatoprotective properties, and its crude bark extract inhibited DPP-4 activity with an IC_50_ of 14.4 µg/mL [98].

### 8.18. Avena sativa

*Avena sativa,* also called the common oat, is a member of the Poaceae family and is widely cultivated in Western China as a staple food. *A. sativa* is considered a functional food due to its health-promoting properties. Oat flour was found to inhibit DPP-4 with an IC_50_ of 0.99 mg/mL [99].

### 8.19. Camellia sinensis

*Camellia sinensis* is a member of the Theaceae family and native to China and Southeast Asia. The principal component of *C. sinensis* is caffeine, which acts as a secondary metabolite. Extract of *C. sinensis* leaves inhibited DPP-4 activity with an IC_50_ value of 227 µg/mL [100].

### 8.20. Vitis thunbergii *var.* taiwaniana

The leaves and fruit of the small-leaf grape (*Vitis thunbergii* var. *taiwaniana*, VTT) are smaller than those of *Vitis vinifera* (standard grape). VTT is used in folk medicine to treat hepatitis, jaundice, diarrhea, and arthritis, and ethanol extracts of the stems and leaves of VTT inhibited DPP-4 activity by 26 and 11%, respectively. The VTT ethanol extracts treatment improved the impaired glucose tolerance of diet-induced obese animals [101].

### 8.21. Prunus amygdalus

*Prunus amygdalus* (also called badaam) is a member of the Rosacease family and is widely distributed in India, especially in the Himalayan region. *P. amygdalus* has several health benefits, which include antioxidative, lipid-reducing, anti-cancer, anti-inflammatory, and immunostimulatory effects. *P. amygdalus* extract inhibited DPP-4 activity with an IC_50_ of 162.9 µg/mL [102].

### 8.22. Ferula assa-foetida

*Ferula assa-foetida* is an herbaceous plant of the Apiaceae family and has a number of medicinal properties. The ethanolic fraction of *F. assa-foetida* inhibited DPP-4 activity by 24.5% [103].

### 8.23. Helichrysum arenarium

*Helichrysum arenarium* is a perennial herbaceous plant of the Asteraceae family and is native to Europe. In European folk medicine, the medicinal properties of this plant are attributed to its choleretic, cholagogic, hepatoprotective, and detoxifying activities. *H. arenarium* methanol extract inhibited DPP-4 enzymatic activity with an IC_50_ of 41.2 µg/mL [104].

**Table 2:** Plants extracts that inhibit DPP-4 and their IC_50_ values.

**S.No.**	**Plant Name**	**Family**	**Plant Part Used**	**Solvent/Extract Types**	**IC_50_ Value**	**Reference**
**1.**	*Urena lobata*	Malvaceae	Roots and leaves	Ethanol	1.65 mg/mL	[76]
**2.**	*Castanospermum austral*	Fabaceae	Seed	Ethanol	13.96 µg/mL	[80]
**3.**	*Fagonia cretica*	Zygophyllaceae	Aerial parts	Crude	38.1 μg/mL	[81]
**4.**	*Hedera nepalensis*	Araliaceae	Aerial parts	Crude	17.2 μg/mL	[81]
**5.**	*Eugenia jambolana*	Myrtaceae	Fruit	Fruit extract	278.94 µg/mL	[82]
**6.**	*Pterocarpus marsupium*	Leguminosae	Heartwood	Heartwood extract	273.73 µg/mL	[82]
**7.**	*Chenopodium quinoa Willd*	Amaranthaceae	Protein hydrolysates	-	0.88 mg/mL	[83]
**8.**	*Allium sativum*	Alliaceae	Garlic bulb	Methanol	70.9 µg/mL	[85]
**9.**	*Pilea microphylla*	Urticaceae	Leaves	Ethanol	520.4 µg/mL	[86]
**10.**	*Mangifera indica*	Anacardiaceae	Leaves	Methanol	182.7 µg/mL	[88]
**11.**	*Psidium guajava*	Myrtaceae	Leaves	Ethanol	380 μg/mL	[92]
**12.**	*Melicope glabra*	Rutaceae	Leaves	Chloroform	169.40 μg/mL	[93]
**13.**	*Emblica officinalis*	Phyllanthaceae	Fruit	Fruit extract	3770 μg/mL	[97]
**14.**	*Berberis aristata*	Berberidaceae	Bark	Methanol	14.4 µg/mL	[98]
**15.**	*Camellia sinensis*	Theaceae	Leaves	Ethanol	227 µg/mL	[100]
**16.**	*Prunus amygdalus*	Rosacease	Seed	Methanol	162.9 µg/mL	[102]
**17.**	*Avena sativa*	Poaceae	Seed	Seed flour	0.99 mg/mL	[99]
**18.**	*Anogeissus latifolia*	Combretaceae	Bark	Water	754 µg/mL	[79]
**19.**	*Aegle marmelos*	Rutaceae	Leaves	Water	790 µg/mL	[79]
**20.**	*Helichrysum arenarium*	Asteraceae	Flowers	Methanol	41.2 µg/mL	[104]

## 9. Natural Phytochemicals

Natural products are an exceptionally rich resource for drug discovery, drug development, and clinical medicine [105]. A summary of natural compounds with DPP-4 inhibitory activity and their corresponding IC_50_ values is provided in Table 3.

### 9.1. Alkaloids

*Coptis chinensis* (family: Ranunculaceae) is a goldthread species native to China. Its rhizomes contain the isoquinoline alkaloids berberine, palmatine, and coptisine and are used in traditional Chinese medicine. In vitro, berberine has a strong DPP-4 inhibitory effect with an IC_50_ value of 13.3 µM [106]. Molecular docking results show 7-deoxy-6-epi-castanospermine derived from *C. australe* inhibited DPP-4 with the same potency as berberine [80].

### 9.2. Flavonoids

The beneficial health effects of fruits and vegetables have been linked to their high flavonoid contents. DPP-4 activity was potently inhibited by anthocyanins isolated from blueberry/blackberry wine blends with an IC_50_ of 0.07 µM [107]. Cyanidin 3-*O*-glucoside inhibited DPP-4 with an IC_50_ of 125.1 µM [108].

Emodin obtained from *Rheum palmatum* inhibited DPP-4 in vitro with an IC_50_ of 5.76 µM [109]. Citrus flavonoids have also been shown to have DPP-4 inhibitory activity; rutin was the most active inhibitor with an IC_50_ of 485 µM [110]. Naringin is abundant in orange peel and has been shown to inhibit DPP-4 in vitro and in vivo and to enhance insulin levels, and thus, is considered an option for the low-cost treatment of diabetes [111].

Rosemary and marjoram extracts were found to inhibit DPP-4 with IC_50_ values of 16 and 29 µM, respectively, and the isolated flavonoids cirsimaritin, hispidulin, and naringenin inhibited DDP-4 activity with IC_50_ values of 0.43, 0.49, and 2.5 µM, respectively [112].

Quercetin is a plant-derived flavonol that has been shown to regulate hyperglycemia and oxidative stress. Molecular docking studies showed that quercetin and galangin bind strongly with DPP-4 and inhibit its activity with IC_50_ values of 4.02 and 40.13 µM, respectively [69,113]. Furthermore, isoquercetin from *Apocynum cannabinum* inhibited DPP-4 with an IC_50_ of 96.8 µM [114].

*Smilax china* plants are found in tropical and temperate regions worldwide, especially in East Asia and North America. Its flavonoids kaempferol 7-*O*-α-l-rhamnoside, vitexin, and rutin were shown to inhibit DPP-4 with IC_50_ values of 20.81, 33.12, and 32.93 µM, respectively [115].

### 9.3. Terpenoids

*Polyathia longifolia* (PL) is used in traditional Indian medicine as a febrifuge and treatment for indigestion. PL has been shown to possess anticancer, antimicrobial, immune-modulating, and anti-ulcer properties [116], and 16-hydroxycleroda-3,13-dien-15,16-olide from PL was found to inhibit DPP-4 activity and to lower BG levels in diabetic mice [117].

The chloroform extract of *Inonotus obliquus* mycelium was also found to inhibit DPP-4. Nineteen compounds were isolated from *I. obliquus* mycelium powders. Molecular docking showed that compounds **5**, **8**, **9**, **14**, and **15** could be the active compounds responsible for DPP-4 inhibition [118].

### 9.4. Phenols

VTT-derived hopeaphenol, (+)-vitisin A, and (−)-vitisin B inhibited DPP-4 with IC_50_ values of 401, 90.75, and 15.3 μM, respectively [101]. Resveratrol, luteolin, apigenin, and flavone potently inhibit DPP-4 with IC_50_ values of 0.6 ± 0.4 nM, 0.12 ± 0.01, 0.14 ± 0.02, 0.17 ± 0.01 μM, respectively, which were lower than the IC_50_ of the diprotin A control (4.21 ± 2.01 μM) [107].

Coumarins are heterocyclic molecules and have been associated with a variety of health benefits, which include antithrombotic, anti-inflammatory, and vasodilatory effects. Coumarins inhibited DPP-4 with an IC_50_ of 54.83 nmol/mL [113]. In a molecular docking study, curcumin was found to bind strongly with DPP-4, and in vitro curcumin inhibited DPP-4 activity by up to 50% [119].

*Macrotyloma uniflorum* (horsegram) is a legume grown mainly in dry regions of Australia, Burma, India, and Sri Lanka and contains high concentrations of myricetin, which has been shown to have anti-hyperglycemic properties. Myricetin also inhibited DPP-4 (IC_50_ of 4.8 μM), and thus, increased serum GLP-1 and insulin levels and ameliorated the manifestations of T2DM [70].

### 9.5. Glycosides

Foods contain a wide range of bioactive molecules, and their different scaffolds and functionalities make them the most important source of possible leads for drug discovery. Virtual screening of a polyphenol-rich food database for potential DPP-4 inhibitors resulted in the identification of chrysin, and an in vitro enzyme assay showed that chrysin inhibits DPP-4 in a concentration-dependent manner [120].

Lentils are the edible seeds of *Lens culinaris* (family Fabaceae), a pulse crop, and have long been grown for human consumption in Europe, the Middle East, Africa, and Asia. Three flavonol glycosides, kaempferol-3-*O*-β-gulcopyranosyl-(1→2)-β-galactopyranosyl-7-*O*-α-rhamnopyranoside, kaempferol-3-*O*-β-gulcopyranosyl-(1→2)-[α-rhamnopyranosyl (1→6)]-β-galactopyranosyl-7-*O*-α-rhamnopyranoside, and robinin (kaempferol-3-*O*-α-rhamnosyl (1→6)-*O*-β-galactopyranoside-7-*O*-α-rhamnopyranoside) isolated from *L. culinaris* seeds were found to inhibit DPP-4 activity in a dose-dependent manner with IC_50_ values of 27.89, 36.52, and 37.01 μM, respectively; molecular docking analysis revealed that these compounds readily fit within the DPP-4 active sites [121].

In Egyptian folk medicine, the herb *Cleome droserifolia* is used to treat diabetes, stomach aches, skin allergies, and open wounds. Five major flavonol glycosides were isolated from an aqueous extract of *C. droserifolia*, and four of these, quercetin-3-*O*-β-d-glucosyl-7-*O*-α-rhamnoside, isorhamnetin-7-*O*-β-neohesperidoside, isorhamnetin-3-*O*-β-d-glucoside, and kaempferol-40-methoxy-3,7-*O*-α-dirhamnoside, showed significant DPP-4 inhibition with IC_50_ values of 0.194, 0.573, 0.345, and 0.281 µg/mL, respectively. In addition, these compounds inhibited aldose reductase and reduced oxidative stress, suggesting their potential use for addressing problems associated with diabetes [122].

The flavonol glycosides (chalconaringenin 2′-*O*-β-d glucopyranoside and aureusidin 6-*O*-β-d-glucopyranoside) derived from *H. arenarium* inhibited DPP-4 with IC_50_ values of 23.1 and 24.3 μM, respectively [104].

**Table 3:** Natural compounds with DPP-4 inhibition and their corresponding IC_50_ values.

**S.No.**	**Phytochemicals**	**IC_50_ Values**	**References**
**1.**	**Alkaloids**		
	Berberine	13.3 µM	[106]
**2.**	**Flavonoids**		
	Cyanidin 3-*O*-glucoside	125.1 µM	[108]
	Anthocyanins	0.07 µM	[107]
	Emodin	5.76 µM	[109]
	Rutin	485 µM	[110]
	Isoquercetin	96.8 µM	[114]
	Cirsimaritin	0.43 µM	[112]
	Hispidulin	0.49 µM	[112]
	Naringenin	2.5 µM	[112]
	Quercetin	4.02 nmol/mL	[113]
	Galangin	40.13 µM	[69]
	Kaempferol 7-*O*-α-l-rhamnoside	20.81 µM	[115]
	Vitexin	33.12 µM	[115]
	Rutin	32.93 µM	[115]
**3.**	**Phenols**		
	Hopeaphenol	401 µM	[101]
	(+)-vitisin A	90.75 µM	[101]
	(−)-vitisin B	15.3 µM	[101]
	Resveratrol	0.6 nM	[107]
	Luteolin	0.12 µM	[107]
	Apigenin	0.14 µM	[107]
	Flavone	0.17 µM	[107]
	Coumarins	54.83 nmol/mL	[113]
	Myricetin	4.8 μM	[70]
**4.**	**Glycosides**		
	Kaempferol-3-*O*-β-gulcopyranosyl-(1→2)-β-galactopyranosyl-7-*O*-α-rhamnopyranoside	27.89 μM	[121]
		36.52 μM	
	Kaempferol-3-*O*-β-gulcopyranosyl-(1→2)-[α-rhamnopyranosyl (1→6)]-β-galactopyranosyl-7-*O*-α rhamnopyranoside	37.01 μM	[121]
	Kaempferol-3-*O*-α-rhamnosyl (1→6)-*O*-β-galactopyranoside-7-*O*-α-rhamnopyranoside	23.1 μM	[104]
	Chalconaringenin 2′-*O*-β-Dglucopyranoside	24.3 μM	[104]
	Aureusidin 6-*O*-β-d-glucopyranoside		
	Quercetin-3-*O*-β-d-glucosyl-7-*O*-α-rhamnoside	0.194 µg/mL	[122]
	Isorhamnetin-7-*O*-β-neohesperidoside	0.573 µg/mL	[122]
	Isorhamnetin-3-*O*-β-d-glucoside	0.345 µg/mL	[122]
	Kaempferol-40-methoxy-3,7-*O*-α-dirhamnoside	0.281 µg/mL	[122]

## 10. Bioactive Peptides

The herbaceous annual plant *Phaseolus vulgaris* (PV) is cultivated globally for its edible dry seeds or unripe berries. Protein fractions derived from PV were found to inhibit DPP-4 activity by 96.7%. In addition, bioactive peptides were isolated from a protein isolate of PV. EGLELLLLLLAG, AKSPLF, and FEELN peptides inhibited DPP-4 more effectively in silico with free binding energy values of −9.8, −9.6, and −9.5 kcal/mol, respectively, than the reference compound sitagliptin (−8.67 kcal/mol) [123]. In another study, protein digests and pure peptides derived from Mexican black and Brazilian Carioca beans inhibited DPP-4 with IC_50_ values ranging from 0.03 to 0.87 mg dry weight/mL [124]. These studies suggest that peptides derived from bean protein isolates can inhibit DPP-4.

*Oryza sativa* L. (rice) bran protein hydrolysates inhibited DPP-4 enzyme with an IC_50_ of 1.45 ± 0.13 mg/mL [125]. When Umamizyme G and Bioprase SP were used to defat and hydrolyze rice bran protein fractions, dipeptides digested with Umamizyme G inhibited DPP-4 with an IC_50_ of 2.3 ± 0.1 mg/mL [126]. Proteins derived from *Amaranthus hypochondriacus* were subjected to simulated gastrointestinal digestion, and this resulted in the formation of bioactive peptides that suppressed DPP-4 in a concentration-dependent manner with an IC_50_ of 1.1 mg/mL [127]. *Glycine max* (soybean) and *Lupinus albus* (lupin) protein hydrolysates contain bioactive peptides, and Soy 1 (IAVPTGVA) and Lup 1 (LTFPGSAED) effectively inhibited DPP-4 with IC_50_ values of 106 and 228 µM, respectively [128]. Gastrointestinal digestion of proteins derived from *Phalaris canariensis* (canary) seeds inhibited DPP-4 by 43.5% [129], and enzymatic digestion of quinoa proteins with papain also resulted in DPP-4 inhibition with an IC_50_ of 0.88 ± 0.05 mg/mL [83]. In a study that used quinoa protein to simulate duodenal digestion, the fraction collected after 120 min of digestion most inhibited DPP-4 with an IC_50_ of 0.84 ± 0.07 mg protein/mL [130].

DPP-4 inhibitory activity was found in peptides isolated from tuna cooking juice hydrolyzed by the enzymes protease XXIII (PR) and orientase (OR). The amino acid sequences of three peptides isolated from PR and OR hydrolysates were PGVGGPLGPIGPCYE (1412.7 Da), CAYEWQRPVDRIR (1690.8 Da), and PACGGFYISGRPG (1304.6 Da), and all three inhibited DPP-4 in a dose-dependent manner [131]. GPAE (372.4 Da) and GPGA (300.4 Da) peptide sequences obtained from Atlantic salmon skin gelatin also potently inhibited DPP-4 [132]. Several peptide sequences in an aqueous *Palmaria palmata* protein extract hydrolyzed with Corolase PP inhibited DPP-4, and three of these peptides (ILAP, LLAP, and MAGVDHI), when purified, potently inhibited DPP-4 with IC_50_ values ranging from 43.40 to 159.37 µM [133]. In addition, peptides obtained from oats, buckwheat, and barley inhibited DPP-4 with IC_50_ values from 0.13 to 8.15 mg/mL, and LQAFEPLR inhibited DPP4 in vitro with an IC_50_ value of 103.5 µM [99]. The bioactive peptides that inhibit DPP-4 and their corresponding IC_50_ values have been listed in Table 4.

## 11. Conclusions

At the molecular level, DPP4 inhibitors work by preventing the degradation of GIP and GLP1, and thus, preserve their endogenous levels and reduce BG levels. Nature has many herbal plants that have long been used to treat diabetes. In this review article, we detail the medicinal plants and their bioactive compounds that inhibit DPP-4 activity. Of these compounds, resveratrol, quercetin, and coumarins are highly effective DPP-4 inhibitors with IC_50_ values in the nanomolar range. Other compounds (flavonoids and phenolics) have the additional benefit of being present in a variety of functional foods. DPP-4 inhibitors improve pancreatic beta cell function and enhance SM cell proliferation. Studies on natural DPP-4 inhibitors offer a powerful means of developing novel treatments for T2DM, and it is hoped this review will help researchers searching for safer, natural DPP-4 inhibitors for the treatment of diabetes.

## Figures and Tables

**Table 1:** Commercial DPP-4 inhibitors.

**S.No.**	**DDP-4 Inhibitor**	**Brand Name**	**Year of Approval**
**1.**	Sitagliptin	Januvia	2006
**2.**	Vildagliptin	Galvus	2007
**3.**	Saxagliptin	Onglyza	2009
**4.**	Alogliptin	Nesina and Vipidia	2010
**5.**	Linagliptin	Tradjenta, Trajenta	2011
**6.**	Anagliptin	Suiny	2012
**7.**	Gemigliptin	Zemiglo	2012
**8.**	Teneligliptin	Tenelia	2012
**9.**	Evogliptin	Suganon	2015
**10.**	Omarigliptin	Marizev	2015
**11.**	Trelagliptin	Zafatek	2015
**12.**	Gosogliptin	Satyor	2016

**Table 4:** Bioactive peptides with DPP-4 inhibition and their corresponding IC_50_ values.

**S.No.**	**Plant**	**Peptide Sequence**	**IC_50_ Value**	**References**
**1.**	*Phaseolus vulgaris*	KTYGL	0.03 mg DW/mL	[124]
		KKSSG	0.64 mg DW/mL	[124]
		GGGLHK	0.61 mg DW/mL	[124]
		CPGNK	0.87 mg DW/mL	[124]
**2.**	*Oryza sativa*	IP	2.3 ± 0.1 mg/ml	[126]
**3.**	*Glycine max*	IAVPTGVA	106 µM	[128]
**4.**	*Lupinus albus*	LTFPGSAED	228 µM	[128]
**5.**	*Palmaria palmata*	ILAP	43.40 µM	[133]
		LLAP	53.67 µM	[133]
		MAGVDHI	159.37 µM	[133]
**6.**	*Avena sativa*	LQAFEPLR	103.5 µM	[99]

## Data Availability

Not applicable.

## Abbreviations

T2DM: type 2 diabetes mellitus, BG: blood glucose, DPP-4: dipeptidyl peptidase-4, GLP-1: glucagon-like peptide 1, GIP: glucose-dependent insulinotropic peptide, SM: skeletal muscles, HFD: high-fat diet, FC: *Fagonia cretica*, MI: *Mangifera indica*, HRS: *Hibiscus rosa-sinensis*, VTT: *Vitis thunbergii var. taiwaniana*, PL: *Polyathia longifolia*, PV: *Phaseolus vulgaris*, PR: protease XXIII, OR: orientase.
